# Distribution and Genetic Variability of Sapoviruses in Africa

**DOI:** 10.3390/v12050490

**Published:** 2020-04-27

**Authors:** Kgomotso Makhaola, Sikhulile Moyo, Lemme P. Kebaabetswe

**Affiliations:** 1 Department of Biological Sciences and Biotechnology, Botswana International University of Science and Technology, Palapye, Botswana; 2 Botswana Harvard AIDS Institute Partnership, Gaborone, Botswana; 3 Department of Immunology & Infectious Diseases, Harvard T.H Chan School of Public Health, Boston, MA 02115, USA

**Keywords:** sapovirus, diarrhea, gastroenteritis, Africa

## Abstract

In this review, we describe the distribution and genetic diversity of sapoviruses detected among humans, animals and the environment in African countries. Databases were searched for studies conducted in African countries and published between Jan 2005 and Mar 2019. Only studies where RT- PCR was used for initial detection were included in the systematic review. We identified 27 studies from 14 African countries with 18 focused on human sapoviruses, two on animal sapoviruses and seven on sapoviruses observed in the environment. Samples. The overall estimated pooled prevalence of human sapovirus infections among symptomatic and asymptomatic individuals was similar at 5.0% (95% Confidence Interval (CI): 3.0–7.0) and 2.0% (95% CI: 1.0–3.0), respectively. In environmental samples sapovirus detection rates ranged from 0% to 90% while in animal studies it was 1.7% to 34.8%. Multiple causes of gastroenteritis, sensitivity of detection method used, diversity of sapovirus strains and rotavirus vaccine coverage rate are some of the factors that could have contributed to the wide range of sapovirus detection rates that were reported. The studies reported human genogroups GI, GII, and GIV, with genogroup GI being the most prevalent. Some potential novel strains were detected from animal samples. Most studies genotyped a small portion of either the capsid and/or polymerase region. However, this is a limitation as it does not allow for detection of recombinants that occur frequently in sapoviruses. More studies with harmonized genotyping protocols that cover longer ranges of the sapovirus genome are needed to provide more information on the genomic characterization of sapoviruses circulating in African countries. Further investigations on animal to human transmission for sapoviruses are needed as inter-species transmissions have been documented for other viruses.

## 1. Introduction

Sapoviruses, whose prototype is Sapporo virus, are single stranded, non-enveloped, positive sense RNA viruses that belong to the *Caliciviridae* family [1,2,3]. Their genome is 7.1 to 7.7 kb in length and has a polyadenylated 3’ end that is essential for viral replication [4]. The 5’ end is linked to VPg that plays a major role in initiation of translation [5,6]. The sapovirus genome consists of two to three open reading frames (ORF) [5]. ORF-1 encodes a large polyprotein that is cleaved into nonstructural proteins and major capsid protein, VP1 [1,2]. ORF-2 encodes minor structural protein VP2 [1]. The function of ORF-3, which is found in some human and bat strains, has not yet been defined [7]. Different methods have been used for the detection of sapovirus that include enzyme-linked immunosorbent assay (ELISA), electron microscopy, next generation sequencing and reverse transcription-PCR (RT-PCR) [3]. RT-PCR is commonly used due to its high sensitivity and specificity [3]. Molecular characterization of sapoviruses is commonly done using a partial VP1 encoding compared to the RdRp region [3,8]. However, determination of new genogroups and genotypes is based on complete VP1 sequences [6,7,9]. By using VP1 sequences, sapoviruses are classified into fifteen genogroups and four of these infect humans (GI, GII, GIV and GV) [2,4,5,10]. Other tentative groups have been identified in bats (GVII to GXVIX) [11]. The VP1 sequence also determines the antigenicity of the virus strains [3,8]. Sapovirus genogroups are further divided into 19 genotypes [6,8]. Human sapovirus genogroups GI and GII comprise of seven and eight genotypes respectively, GIV includes a single genotype and GV can be divided into two genotypes [2,12]. Strains of sapovirus with discordant grouping between the VP1 encoding region and the RdRp have been classified as recombinants [5,13,14,15]. Recombination was shown to occur most frequently in the RdRp-VP1 junction [13,16,17]. However recombination events have also been reported in the NS3-NS4 junction [17]. The RdRp-VP1 recombination can alter the pathogenicity and virulence of the resulting strains [8,14,17]. Co-infection with different sapovirus strains has been reported and represents a precondition enabling recombination to occur [14]. Sapoviruses have been detected in humans and various animals including pigs, minks, dogs, sea lions, chimpanzees, bats and rats (Table 1) [1,2,3,4,5,10,18,19,20,21]. Sapoviruses have also been detected in environmental samples (river water, wastewater and sewage) posing a risk to populations exposed to contaminated water [7,22].

Sapoviruses are the causative agents of viral gastroenteritis and their most common symptoms are vomiting and diarrhea [5]. Globally, diarrhea is common among children, and it is identified as the second leading cause of death among children under 5 years with an estimated 2 million deaths occurring annually [23]. Diarrhea has been identified as the leading cause of morbidity and deaths among HIV-infected children [24,25]. The Sub-Saharan Africa region is one of the regions with the highest prevalence of HIV [26]. Other enteric viruses like rotavirus have been shown to increase the burden of viral gastroenteritis among HIV-infected children [24]. Sapoviruses have been identified in both sporadic and outbreak cases of acute gastroenteritis [2,8] and have a higher prevalence in children than in adults [2,19]. Sapovirus-related viral gastroenteritis is often self-limiting and generally resolves within 3–4 days. However, cases of individuals showing symptoms longer and with more than average severity have also been reported especially among the immune-compromised [3,8,27]. Currently vaccines against sapovirus are not available and treatment is mainly targeted at resolving symptoms [3,28].

Sapovirus transmission occurs through the fecal-oral route either via contaminated food, water, surfaces or by direct contact with infected individuals [18,20]. Since sapoviruses have been detected in rivers, sewages and treated water [21], access to clean drinking water is essential for prevention and control. Shedding of the virus by asymptomatic individuals has also been documented [29], indicating that proper food handling practices are also important for prevention and control of infections.

The objective of this review is to understand the distribution and molecular diversity of sapoviruses circulating in African countries among humans, animals and in the environment. In reviewing human infections, factors like age, seasonality, and co-infections were also evaluated. Where data on the HIV status of participants were available, their impact on sapovirus infection was also assessed. 

## 2. Literature

### 2.1. Search and Selection Strategy

A systematic literature review of sapovirus studies from African countries published in peer reviewed journals was performed. Electronic databases PubMed, Medline, and Google Scholar were searched using key words “sapovirus”, “caliciviruses”, “gastroenteritis”, “Africa”, and “name of country” alone and in various combinations. To ensure that studies that do not appear in major electronic databases were not missed, Rayyan [30] and Google Search were also used. 

Studies from African countries published between January 2005 and March 2019 that described sapovirus infections among humans, animals and environmental samples were included in this review. Only studies where sapovirus initial detection was carried out by RT-PCR were included. For genotyping, all studies that used any region of the capsid and or polymerase were included.

### 2.2. Data Extraction and Analysis

The following variables were extracted from each of the selected studies where available: study reference, publication date, period and duration of the study, clinical symptoms, type of gastroenteritis (sporadic, outbreak), specimen type (human stool, animal stool, environmental sample), study population (symptomatic, asymptomatic), age group, diagnostic method used, prevalence, seasonality, co-infections, genogroups and genotypes identified. In assessing seasonality, only studies with duration of 12 months or more were included. We estimated the prevalence of sapovirus (the proportion of cases) for each study. We also estimated pooled sapovirus prevalence rates by age category and symptoms presentation with exact 95% confidence intervals (CI) using STATA version 15.1. In order to account for heterogeneity we also conducted random-effects meta-analyses to pool the prevalence of the sapovirus by age grouping and presentation of symptoms. We assessed the variability across studies using I^2^ statistics and assessed for heterogeneity using forest plots (with prevalence estimates with 95% intervals. I^2^ describes the percentage of total variation due to inter-study heterogeneity. STATA version 15.1 (SataCorp, College Park, Texas, USA) was used for the analysis. 

All sequences available from the reviewed studies were retrieved from National Center for Biotechnology Information (NCBI) sequence databases. For analysis of genetic variation among isolated strains sequences from each study were used to construct phylogenetic trees using either BEAST V 1.7 [31] or MEGA V 7.0 [32]. The following strains were used as reference sequences: GI.1 (X86560, MK111630, MG012400, AY237422); GI.2 (MH541039, MN164924, AB602119, EU124657); GI.3 (AF194182, AB622459); GI.4 (AJ606693; GI.5 (KM282596, AY538716, AJ606698); GI.6 (KX866362, FJ65744); GI.7 (KT276532, AB181133); GII.1 (MG012407, AJ271056); GII.2 (KT276556, AY237420); GII.3 (MN164975, AB689808); GII.4 (AB29084, KP067444); GII.6 (MK246750); GII.7 (AB630067); GII.8 (HM590581, KT30674); GIII (AFI82760, AY425671.1); GIV.1 (DQ104357, HM214146, AF435814.1); GV.1 (AY646856); GV.2 (AB775659); GVI (KJ508818.1); GVII (KX000384.1); GVIII (KC309417.2); GIX (KC309418.3); GXI (LC215902.1); GXII (KX000385.1); GXIII (JN387134.2); GXIV (JN899075.1).

## 3. Sapovirus in African Countries

**Figure 1:**
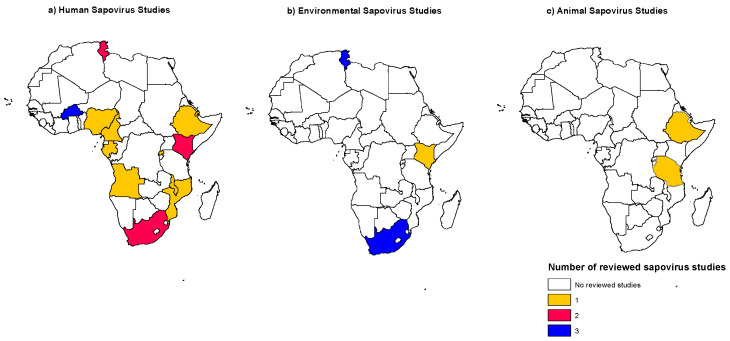
Map of Africa showing the countries where (**a**) human (**b**) environmental, and (**c**) animal sapovirus studies were published, Jan 2005–Mar 2019. The different colors indicate the number of studies published per country, while white indicates no data available.

After reviewing data based on the inclusion criteria, 27 studies from 14 African countries (Angola, Burkina Faso, Cameroon, Djibouti, Ethiopia, Gabon, Kenya, Malawi, Mozambique, Nigeria, South Africa, Rwanda, Tanzania and Tunisia) were included in the review. Among the 27 eligible studies, 18 (66%) were on human sapoviruses, two (7%) were on animals whereas seven (26%) were on sapoviruses in environmental samples (river water, wastewater and sewage); Figure 1, shows the map of various countries where the studies were conducted. 

### 3.1. Distribution of Human Sapovirus Infections

Table 2 summarizes human sapoviruses studies from the 13 countries that were reviewed [33,34,35,36,37,38,39,40,41,42,43,44,45,46,47,48,49,50]. While age groups varied among the studies, the majority, 89% (16/18), included children less than 5 years [34,35,36,37,38,39,40,41,42,43,44,45,46,47,48,49,50]. Relatively few sapovirus studies were conducted in Africa during the review period. However there has been an increase over the last four years, with 68% (13/19) of the studies published between 2015 and 2019 [34,36,37,38,39,41,42,43,44,48,49,50,51]. The method of detection was either real-time RT-PCR or RT-PCR with primers that targeted either the VP1 region [36,46]; RdRp region [35,40,45,47] or the RdRp-VP1 junction region [33,37,38,39,42,43,48,49,50]. The reported studies varied in sample size and duration, and the rate of detection among symptomatic individuals ranged from 0% in Nigeria to 19% in Angola [34,50]. Differences in detection rates were not only observed across countries, but also among studies conducted in different regions of the same country. For example, a study conducted in Kenya reported sapovirus detection rates of 4% in Lwak district and 6% in Kibera district [43], while the other study also conducted in Kenya reported a sapovirus detection rate of 10.8% among symptomatic individuals [42]. Differences in sapovirus detection rates were also observed over time. In Burkina Faso, a study conducted among symptomatic under-five-year-old children between 2009 and 2010 reported a sapovirus detection rate of 18% [38] and another one conducted between 2011 and 2012 reported a sapovirus detection rate of 10.3% [49].

### 3.2. Age and Sapovirus Distribution

**Figure 2:**
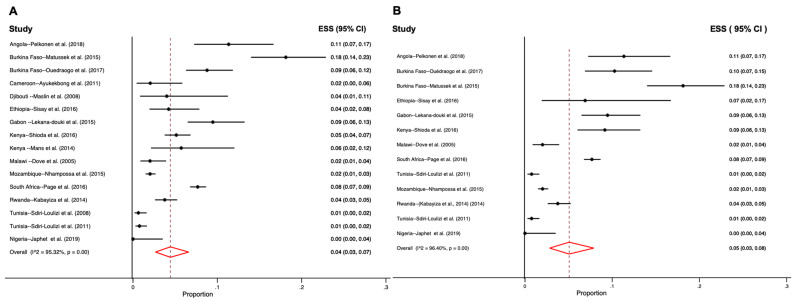
Forest plots for pooled prevalence by age estimated with random-effects meta-analyses (**A**) all ages and (**B**) under five years of age. (ESS—estimated effect size or proportion of cases; I^2^—heterogeneity statistic; CI—confidence interval).

Figure 2, shows pooled stool positivity rate by age categorization estimated with random-effects meta-analyses. It shows that substantial heterogeneity exists, and the I^2^ for these studies is greater than 80%. Seventeen of the 18 reviewed studies included prevalence data [33,34,35,36,37,38,39,40,41,42,43,44,45,46,47,49,50,51], and were used to compute pooled stool positivity rate which was 4.0% (95% CI 3.0–7.0) across all ages and 5.0% (95% CI 3.0–8.0) for under-five-year-olds. Studies that did not have data that allowed for disaggregation by the under-five age category were excluded from this calculation. Three out of the four studies that reported a prevalence of greater than 10% were conducted among children less than five years old. In Angola this prevalence was 19% and two studies in Burkina Faso reported 10.3% and 18%, respectively [34,38,39]. 

**Figure 3:**
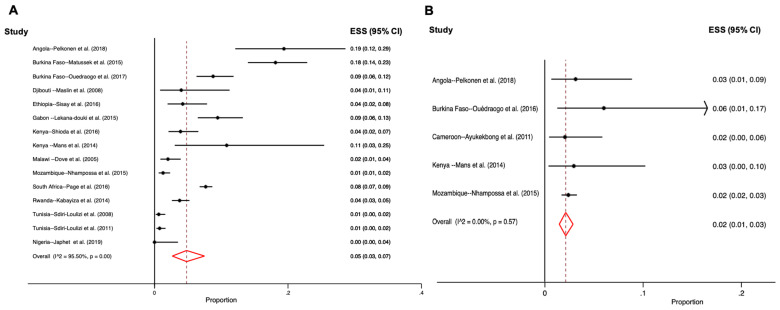
Forest plots for meta-analysis of sapovirus prevalence among (**A**) symptomatic, (**B**) asymptomatic individuals. (ESS—estimated effect size or proportion of cases; I^2^—heterogeneity statistic; CI—confidence interval).

Among symptomatic and among asymptomatic participants the estimated pooled stool positivity rates were similar at 5.0% (95% CI 3.0–7.0) and 2.0% (95% CI 1.0–3.0), respectively (Figure 3). While some studies reported only mild cases of diarrhea and vomiting, others reported more severe cases where individuals were hospitalized [35]. Only one study reported cases of bloody diarrhea associated with sapovirus/HIV co-infection [44].

### 3.3. Sapovirus and Coinfections

Sapoviruses often occurred with other enteric bacteria like *Cryptosporidium*, *E. coli* and *Shigella* [36,44] and other viruses that included rotavirus, norovirus and adenovirus [49]. One study showed that children with diarrhea and sapovirus infection were likely to be infected with more than one enteric pathogen [44]. Three studies enrolled participants with known HIV status [33,42,44]. A study in Cameroon reported higher detection of enteric viruses among asymptomatic children who were HIV negative compared to asymptomatic adults who were HIV positive [33]. One study in South Africa reported that 37.5% (3/8) of children co-infected with sapovirus and HIV died [44]. These co-infected children were also likely to have bloody stools with mixed pathogen infections. A study from Kenya did not indicate any susceptibility to sapovirus infections due to an individuals’ HIV status [42].

### 3.4. Seasonality of Human Sapovirus Infections

Sixty seven percent (12/18) of the studies conducted lasted 12 months or more and were used to assess the seasonality of the infections [35,36,37,38,40,43,44,45,46,47,48,50]. These studies were three from Southern Africa, four from East Africa, two from North Africa and three from West Africa. Most of the studies did not show any clear seasonal patterns as infections occurred all year round. However, in North Africa (Tunisia) one of the two studies showed a winter peak [45] but the overall prevalence in the study was low at 0.8%. One study conducted in Burkina Faso, West Africa, showed a peak in the cold dry season [39]. The study in Malawi, Southern Africa showed a slight peak in the rainy season [35].

### 3.5. Genetic Characterization of Human Sapoviruses 

Molecular data were available for 7 out of the 18 studies conducted in six countries of Burkina Faso, Ethiopia, Kenya, Malawi, South Africa and Tunisia [35,38,42,45,48,49,52]. There was wide genetic diversity of human sapoviruses detected in these studies. Based on the constructed phylogenetic trees that used 240 bp sequences of the VP1 region and 183 bp of RdRp region (Figure S1) from human sapovirus studies, the sapovirus strains clustered into three genogroups of GI, GII and GIV. Genogroup GI (GI.1 to GI.7) constituted most of the infections at 48%, followed by GII (GII.1, GII.2, GII.4, GII.5, GII.6, GII.7) at 31% and GIV at 21%. GI and GII were detected in seven countries [35,38,41,42,45,48,49]. Genogroup GIV was isolated in Burkina Faso and South Africa [38,48].Nineteen genotypes were identified and GIV.1 was the most predominant at 21% followed by GI.2, GI.1 and GII.1 at 20%, 18% and 17% respectively. The RdRp-VP1 sequences were not long enough to allow for detection of recombinant strains.

Nineteen genotypes were identified and GIV.1 was the most predominant at 21% followed by GI.2, GI.1 and GII.1 at 20%, 18% and 17% respectively. The RdRp-VP1 sequences were not long enough to allow for detection of recombinant strains.

### 3.6. Sapovirus Distribution in the Environment

**Figure 4:**
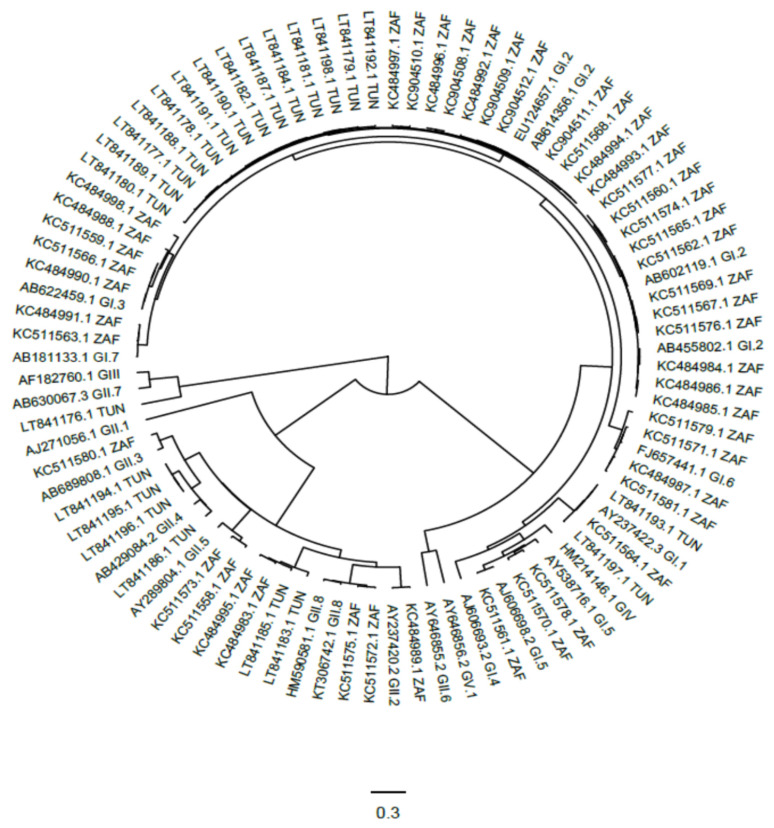
A phylogenetic tree of environmental sapovirus, 260 bp of partial VP1 region, sequences. Reference sequences are annotated with accession number and genotype while sequences from reviewed studies have accession number and country code. The evolutionary history was inferred by using the (BEAST v 1.10.4) Bayesian method, GTR model.

Seven studies from three countries, Kenya, South Africa, and Tunisia reported on sapovirus strains that were isolated from environmental samples, which comprised wastewater, river water and sewage [21,53,54,55,56,57,58] (Table 3).These studies were conducted between 2003 and 2010 and ranged in duration from nine to twelve months. All of the studies were conducted in countries where human sapovirus studies had also been conducted allowing comparison of genotypes isolated from the environment to those circulating in the communities. In one study, sapovirus was not detected in sewage or wastewater [57]. Primers used in this study targeted the RdRp region. However, another study showed that sapovirus detection rate was as high as 90% in rural river water and sewage [53] and these used primers that targeted the RdRp-VP1 junction. In some instances treated wastewater and river water had high concentrations of sapoviruses with prevalence reaching more than 50% [56]. The reported data showed that not only were sapoviruses detected in higher concentrations but there was a wide diversity of sapovirus strains circulating in river water and water bodies in Africa. Three studies from South Africa and two from Tunisia had molecular data available where genotyping was done using either the partial capsid or partial RdRp region [21,54,55,56,58]. Based on the phylogenetic analysis the sapovirus strains clustered in three genogroups GI (GI.1, GI.2, GI.3.GI.5, GI.6, GI.7); GII (GII.1, GII.2, GII.3, GII.4, GII.5, GII.8) and GIV, (Figure 4 and Figure S2). Genogroups GI and GII were isolated in both countries but GIV was only isolated in Tunisia. Two sapovirus strains isolated in Tunisia could not be assigned to any genotype [58] and these were identified using primers targeting partial RdRp.

### 3.7. Animal Sapovirus Infections

Two animal sapoviruses studies from Ethiopia and Tanzania [52,59] were reviewed (Table 4). The animals that were studied included pigs [52], spotted hyenas, African lion and bat eared fox [59]. The detection rate of sapovirus ranged from 1.7% among pigs in Ethiopia [52] to 34.8% among spotted hyenas in Tanzania [59]. In both studies, sequencing was done using primers that target the RdRp region. In Ethiopia, one of the sapovirus strains isolated from pigs [52] was identified as genogroup GIII and another one could not be assigned (Figure S3). Sapovirus strains detected in spotted hyenas, African lions, and bat eared fox from Tanzania clustered in monophyletic group and these could be a potential new group.

## 4. Discussion

Viral gastroenteritis is a public health concern, with high morbidity and mortality particularly in the under 5 years age group [60]. Sapoviruses are recognized as one of the causative agents of viral gastroenteritis. Therefore, it is important to understand their distribution and molecular characterization in each region and country to contribute towards prevention and management strategies. There is no treatment for sapovirus disease, and prolonged shedding of the virus even after cessation of symptoms has previously been reported [20]. In this review, the pooled sapovirus prevalence among symptomatic cases, 5.0% (95% CI 3.0–8.0), and among asymptomatic, 2.0% (95% CI 1.0–3.0), were not statistically different. However, the detection rates observed in the reviewed African studies is consistent with what has been reported in other countries [9,19,60,61]. Since sapovirus has been identified as one of the causative agents of gastroenteritis [3], one would have expected rates among symptomatic individuals to be significantly higher than those among asymptomatic. One contributing factor could be the multiple causes for gastroenteritis that include bacteria and other viruses predominantly rotavirus and norovirus [61]. This was reported by one study from Nigeria that isolated rotavirus and norovirus strains and no sapovirus from symptomatic individuals [50]. In the reviewed studies, a wide range of sapovirus stool positivity rates varied from 0% in Nigeria, West Africa [50] to 19% in Angola, Southern Africa [34]. These ranges were similar to those reported in other countries outside Africa: Thailand 1.1% [19], Nicaragua 17% [29]; Peru 12.4% [2] and Philippines 7% [8]. 

The differences observed in sapovirus prevalence rates may also be due the sensitivity of the detection methods used in these studies. RT-PCR methods for the detection of sapoviruses have limitations even though they have higher sensitivity compared to other methods such as ELISA [3,4]. Amplification that targets the more conserved region between the polymerase and capsid has been shown to detect diverse genogroups compared to those that use either capsid or polymerase region only [3,8]. Most of the reviewed studies [38,42,49] that reported sapovirus detection rate of at least 10% were where the initial detection method used primers that targeted the conserved RdRp-VP1 region. Primers targeting the RdRp-VP1 region are able to amplify multiple sapovirus genotypes [4]. One study where primers targeting this region were used reported only six positive sapovirus cases belonging to the five sapovirus genotypes [42]. Another study in South Africa, using a similar method identified at least thirteen different sapovirus genotypes [48], indicating the sensitivity of this method in amplification of sapovirus. However, limitations were observed in some of the studies where primers targeting the partial RdRp were used to amplify the sapovirus strains [35,41,49,58,59]. This often resulted in misclassification of the sapovirus strains or inability to confidently declare new genotypes. For example, a study conducted in Malawi [35] initially identified the isolated sapovirus strain as a GIII, and further analysis classified this as a GII.1 (Figure S2). Some sapovirus studies conducted in Tunisia identified strains that did not cluster with known sapovirus genotypes [58,59]. While these could be potential new genotypes they could not be classified as such since new genotypes are normally defined based on the complete VP1 sequence [3]. These reports indicate the need for standardized and more sensitive methods for the detection of sapovirus.

The coverage rate of rotavirus vaccination may also contribute to the differences in sapovirus prevalence among the reviewed African countries. It has been reported that where rotavirus vaccination coverage is high, norovirus and sapovirus infections are prominent [29] and with rotavirus vaccination coverage of greater than 95%, sapovirus infections were the second major cause of viral gastroenteritis following norovirus [29]. This information underscores the need to understand the burden of sapovirus in each country especially in countries where rotavirus vaccine coverage is high. Between 1998 and 2014 when most of the reviewed studies were conducted, rotavirus vaccination coverage among African countries ranged from 0% to more than 95% [62,63] and this can contribute to the differences in the observed sapovirus detection rates among symptomatic individuals. It was observed that detection rates of sapovirus infections also differed in regions of the same country [43], where the same factors would contribute to prevalence.

Globally, gastroenteritis continues to affect many people, especially children and the elderly. The pooled prevalence on sapovirus detection among symptomatic children under 5 years was 5.0% (95% CI 3.0–8.0), which was comparable to those reported in other regions, and confirmed sapoviruses as one of the many causes of gastroenteritis among children under 5 years [8,29,60]. Though some of the studies included all age groups, data on the impact of sapovirus infections among the elderly were scarce. Gastroenteritis is also a common illness of diverse etiology among the elderly especially those in nursing homes [64]. Understanding the impact of sapovirus to this health problem can contribute to its management and prevention among this age group.

The findings of generally milder symptoms of diarrhea, vomiting, and dehydration that were associated with sapovirus infections [49,65] is comparable to what other studies have described in other regions outside Africa [29,60,66]. However, severe sapovirus symptoms are not uncommon, and have often been associated with GI infections [29]. A study from South Africa [44], reported more severe symptoms that included those in which bloody stool and sapovirus was detected in 11.4% (9/79) of those who died, with three of them being HIV infected. The GI.2 was also the most common strain in this cohort and could have contributed to the severe symptoms. Moreover, lack of access to sanitation was observed to increase rates of sapovirus infection especially among HIV-infected children. Control measures for the prevention of sapovirus infections should focus more on maintaining good personal, food and environmental hygiene.

There is an indication that different weather conditions can affect sapovirus infection. Some studies have reported seasonal patterns of sapovirus infections with most infections from sporadic cases occurring in the cold season [3,67]. However, other studies have reported infections that occurred all year round [35,45]. The reviewed African countries fall in regions of different weather conditions and these differences are sometimes observed within a country. A study in Tunisia showed that infections peaked in winter, similar to studies in Japan and Iran [67,68]. However, another study in Tunisia did not show any seasonal patterns [47]. Furthermore, a study conducted in Burkina Faso showed infection peaks in the cold and dry season [49]. Nonetheless, this study only lasted for 10 months [49]. Prevention and management efforts that include, among others, long-term surveillance programs for all common etiological agents of gastroenteritis in each country can provide the necessary information for its control and management.

Sapovirus as an RNA virus undergoes frequent mutations leading to the emergence of new strains that have altered antigenicity and can persist over time leading to more disease outbreaks [3]. Among the eight studies that conducted molecular characterization of sapoviruses, the detected genogroups, GI, GII and GIV have also been identified in outbreaks in other continents [4,67,68]. On average GIV was the most prevalent genogroup isolated mainly in South Africa between 2009 and 2013 [48]. In other regions of USA, Japan, and Europe, this strain was most prevalent in 2007 [3]. Initially, classification of sapovirus was not standardized leading to some strains being wrongly classified. An example is a study conducted in 2005 in Malawi among hospitalized children, which reported isolation of eight strains of sapovirus, and all of them were classified as GIII [35]. GIII is commonly isolated in pigs [1,18] and its presence in humans has not been reported elsewhere. Sapoviruses like most RNA viruses evolve through recombination to improve their fitness and evade the host immune system. The use of whole genome sequences or those that cover the ORF1/ORF2 junction are needed to detect the presence of recombinants. Studies that provide sapovirus whole genome analyses are needed in African countries to add to the body of knowledge in this area, especially the discovery of novel strains.

Sapovirus strains have also been detected in environmental samples of river water, treated and untreated sewage and very often strains isolated in the environment reflect those circulating in the surrounding communities [69]. In these studies, sapovirus strains detected in the wastewater bodies were similar to those identified on other continents [22,54,69]. There was correlation in the frequency of sapovirus detections in the environment and the prevalence of human infections, for example, in Tunisia where human sapovirus studies reported very low detection rates of 0.8% and 1% [45,47]. Studies conducted on sewage samples did not detect any sapovirus [57]. Another study conducted in Tunisia in waste water identified potential new genotypes belonging to genogroups II [58] and similar genotypes had previously been detected among children in USA [70]. In South Africa, genotypes that were isolated in human studies were also detectable in river water, treated and untreated wastewater [48,55,56]. The stability of sapoviruses in different environmental conditions influences their transmission back into the community. In the different countries that were reviewed, living conditions such as access to clean water and cultural practices in food handling vary in different parts of a country, and can contribute to the different patterns observed in the distribution of sapoviruses.

While most mammalian sapoviruses belong to particular genogroups not infecting humans, strains that genetically resemble those of humans have been identified [4]. Thus, these could possibly serve as reservoirs for human infections or vice-versa [5]. Some of the factors that have been suggested to enhance animal to human transmission include broad host range and infection of wild animals [71]. As shown in Table 1, sapoviruses have a wide host range including wild animals, making them a potential candidate for human-animal transmission.

In conclusion, this review showed that human sapoviruses circulating in African countries are genetically diverse, with stool positivity rates varied from 0% to 19%. Sapovirus detection rates in environmental samples ranged from 0% to 90% while in animal studies it was 1.7% to 34.8%. Potential new sapovirus strains have also been identified and these can contribute to broader understanding of sapovirus evolution. From the reviewed data, it is evident that studies in Africa can contribute to the discovery of novel sapovirus strains especially in areas with a high population of animals. Moreover, due to emerging viruses or mutations, more studies need to be undertaken that characterize longer sections of the genome.

## Figures and Tables

**Table 1:** Distribution of sapovirus genogroups by species.

Species	Genogroup	References
Humans	GI, GII, GIV, GV	[2,4,18,19,20]
Dogs	GXIII	[4]
Pigs	GIII, GV, GVI, GVII, GVIII, GIX, GX, GXI	[1,3,10]
Bats	GXIV, (GXVIII, GXVIX-tentative)	[4,11]
Mink	GXII	[4]
Chimpanzees	GI	[4]
Sea lions	GV	[4]
Rats	GII, GXV	[4]

**Table 2:** Summary of reported studies on human sapovirus infections in Africa.

Reference	Country	Study Period; Duration	Prevalence	Age (Years)	Sapovirus Detection Method	Sequencing Region and Nucleotide Position	Identified Genotypes (Based on Partial VP1 Sequence)	Identified Genotypes (Based on Partial RdRp Sequence)
[34]	Angola	Dec 2013 to Aug 2014; 8 months May to Oct 2014; 6 months	All 11% (22/194) Symptomatic 19% (19/98) Asymptomatic 3% (3/96)	<5	Real-time RT PCR ^a^	n/a	n/a	n/a
[49]	Burkina Faso	Nov 2011 to Sep 2012; 10 months	Symptomatic 10.3% (27/263) Asymptomatic 6% (3/50)	<5	Real-time RT-PCR ^b^	Partial RdRp ^l^ nt 4366–4884 *	n/a	GII.1(3) GII.2(2) GIV.1(1) Unassigned (3)
[38]	Burkina Faso	May 2009 and Mar 2010; 12 months	Symptomatic 18% (56/309)	<5	Real-Time RT-PCR ^c^	Partial VP1 ^h^ nt 5083–5516 *	GI.1(12) GI.2(6) GI.4(4) GII.1(1) GII.2(2) GIV.1(2)	n/a
[39]	Burkina Faso	Nov 2011 to Sep 2012; 10 months	Symptomatic 10.3% (27/263) Symptomatic 6.5% (11/170)	<5 5–89	Real-Time RT-PCR ^b^	n/a	n/a	n/a
[33]	Cameroon	Oct to Dec 2009; 2 months	Asymptomatic 2.04% (3/147)	5–75	Real-Time RT-PCR ^c^	n/a	n/a	n/a
[40]	Djibouti	Sep 2002 to Feb 2004; 15 months	Symptomatic 4% (3/75)	>15	RT-PCR ^d^	n/a	n/a	
[41]	Ethiopia	Jun to Sep 2013; 3 months	Symptomatic 4.2% (9/213)	All	RT-PCR ^e^	Partial RdRp ^e^ nt 4327–4656 **	n/a	GII.1
[37]	Gabon	Mar 2010 to Jun 2011; 15 months	Symptomatic 9.5% (30/317)	<5yrs	multiplex real-time RT-PCR ^f^	n/a	n/a	n/a
[43]	Kenya	Jun 2007 to Oct 2008; 16 months Oct 2006 to Feb 2009; 28 months	Symptomatic 4% (13/334) Lwak District 6% (31/524) Kibera District	All	Real-time RT-qPCR ^c^	n/a	n/a	n/a
[42]	Kenya	Feb 1999 to Jun 2000; 16 months	Symptomatic 10.8% (4/37) Asymptomatic 2.9% (2/68)	<14	Real-Time RT-PCR ^c^	Partial VP1 ^m^ nt 5098–5878 *	GI.2(2) GI.6(1) GII.1(1) GII.2(2) GII.4(1)	n/a
[35]	Malawi	Jul 1998 to Jun 1999; 12 months	Symptomatic 2% (8/398)	<5	RT-PCR ^g^	Partial RdRp ^g^ nt 4354–4684 *	n/a	GII.1
[36]	Mozambique	Dec 2007 to Oct 2011; 46 months	Symptomatic 1.3% (10/784) Asymptomatic 2.4% (38/1595)	<5	Multiplex RT-PCR ^h^	n/a	n/a	n/a
[50]	Nigeria	Aug 2012 to Dec 2013; 16 months	Symptomatic 0% (0/103)	<5	Real-time RT-qPCR ^i^	n/a	n/a	n/a
[44]	South Africa	Apr 2009 to Dec 2013; 44 months	Symptomatic 7.7% (238/3103)	<5	Real time RT-PCR ^j^	n/a	n/a	n/a
[48]	South Africa	Apr 2009 to Dec 2013; 44 months	n/a	<6	Real time RT-PCR ^j^	Partial VP1 ^n^ nt 5159–5498 *	GI.1(27) GI.2(45) GI.3(8) GI.5(8) GI.6(4) GI.7(2) GII.1(29) GII.2 (7) GII.3(14) GII.4(18) GII.5(7) GII.6(2) GII.7(2) GIV.1(49)	n/a
[46]	Rwanda	Nov 2009 to Jun 2012; 30 months	Symptomatic 3.8% (33/879)	<5	Real-time RT-PCR ^k^	n/a	n/a	n/a
[45]	Tunisia	Jan 2003 to Apr 2007; 39 months	Symptomatic 0.8% (6/788)	<2	RT-PCR ^l^	Partial RdRp ^l^ nt 4366–4884 *	n/a	GI.1(1)
[47]	Tunisia	Jan 2003 to Jun 2005; 29 months	Symptomatic 1% (4/632)	<12 years	RT-PCR ^l^	n/a	n/a	n/a

^a^ Not available; ^b^ SaV124F/SaV1Fa/SaV1245R SaV124TP; ^c^ SaV124F/SaV1F/SaV5/SaV1245R SaV124TPFAM/SaV5TPFAM; ^d^ SR80/p110; ^e^ PEC66/PEC65; ^f^ sapo.fwdA/sapo.fwdA/sapo.rev sapo.probeA/sapo.probeB/sapo.probeC/sapo.probeD; ^g^ P289/P290; ^h^ SLV5749/SLV5317; ^i^ SV56A/SV56B/SV5/Sav1245 RSVTM1 VIC/SAV5 TP VIC; ^j^ CU-SV-F1/CU-SV-F2/CU-SV-R/CU-SV-Probe; ^k^ Forward 1/Reverse Probe VIC; ^l^ SR80/NVP110; ^m^SaV124F, SaV1F, SaV5F, SaV1245R, SV-F11/SV-R1; ^n^ SV-DS5, SV-DS6 SV-F13, SV-F14, SV-DS3, SV-DS4, SaV1245Rfwd, SV-DS5, SV-DS6; * Primer location based on sapovirus strain Manchester X86560; ** Primer location based on sapovirus strain Cowden porcine enteric virus (AF182760).

**Table 3:** Summary of reported studies of environmental sapovirus in Africa.

Ref	Country	Study Period; Duration	Prevalence	Specimen Source	Sapovirus Detection Method	Sequencing Primers and Sequenced Region	Identified Genotypes -Based on Partial VP1 Sequence (# of Samples)	Identified Genotypes -Based on Partial RdRp Sequence (# of Samples)
[53]	Kenya	May 2007 to Feb 2008; 9 months	90% (9/10) 14.3% (1/7)	urban and rural river water, sewage	Real-time RT-PCR ^a^	n/a	n/a	n/a
[56]	South Africa	n/a	80% (8/10)	wastewater treatment works and affected wastewater bodies	Real-time RT-qPCR ^b^	partial VP1 ^e^ nt 5157–5591 and 5159–5498 *	GI.2(6/6)	n/a
[54]	South Africa	Aug 2010 to Dec 2011; 16 months	73% (37/51)	wastewater	Real-time RT-qPCR ^b^	Partial VP1 ^f^ nt 5159–5498 *	GI.2 (8) GI.3 (3) GI.6 (1) GI.7 (1) GII.1 (1) GII.2(1)	n/a
[55]	South Africa	Jan 2009 to Dec 2010; 23 months	41% (21/51) in 2009 58% (27/48) in 2010	river water	Real-time RT-PCR ^a^	Partial VP1 ^f^ nt 5159–5498 *	GI.1(1) G1.2(9) GI.3(2) GI.5(3) GI.7(1) GII.3(1) GII.5(1) GII.8(3)	n/a
[21]	Tunisia	Dec 2009 to Dec 2010; 12 months	39.9% (87/218) All 56% (61/109) Untreated 23.9% (26/109) Treated	wastewater treatment plants	Real-time RT-PCR ^c^	Partial VP1 ^g^nt 5159–5591 *	GI.1(2) GI.2(15) GII.1(2) GII.4(4) GII.8(1)	n/a
[57]	Tunisia	Jan 2003 and April 2007	0% (0/250) sewage	sewage samples	RT-PCR ^d^	n/a	n/a	n/a
[58]	Tunisia	Jan-Dec 2011; 12 months	untreated 1 to 17% treated	Waste water	Real-time RT-PCR ^c^	Partial RdRp ^d^ nt 4366–4884 *	n/a	GI.3(1) GIV.1(1) Could not assign (2)

^a^ CU-SV-F1/CU-SV-F2/CU-SV-R/CU-SV-Probe; ^b^ CU-SV-F1, CU-SV-F2 and SaV1245R; ^c^ SaV124F/SaV1245R, SaV124TP; ^d^ SR80/NVP110; ^e^ SaV124F, SaV1F, SaV5F, SV-R14 and SV-R14, SaV1245Rfwd, SV-R2; ^f^ SV-DS5, SV-DS6, SV-F13, SV-F14, SV-DS3, SV-DS4, SaV1245Rfwd, SV-DS5, SV-DS6; ^g^ SV-R13, SV-R14, SaV1F, SaV5F, SaV124F, SV-R2, 1245Rfwd; * Primer location based on sapovirus strain Manchester X86560.

**Table 4:** Summary of reported studies on animal sapovirus in Africa.

Reference	Country	Study Period; Duration	Prevalence	Animal Species	Initial Detection	Sequencing Primers and Sequenced Region	Identified Genotypes (Based on Partial RdRp Sequence)
[52]	Ethiopia	Jan–Sep 2013; 9 months	1.7% (2/117)	swine	RT-PCR ^a,b^	Partial RdRp ^a^ nt 4327–4656 * Partial RdRp ^b^ nt 4568–4884 **	GIII (1/2) (1/2) could not be assigned
[59]	Tanzania	2001–2012; 11 years	34.8% (171/514) 33.3% (3/9) 22.2% (2/9)	Spotted hyenas African lion Bat eared fox	RT-PCR ^c^	partial RdRp ^d^	20/20 clustered together but could not be assigned (potential novel strains)

^a^ PEC66/PEC65; ^b^ p290/p110; ^c^ P289/P290; ^d^ Cali2R/90R; * Primer location based on sapovirus strain Cowden porcine enteric virus (AF18276); ** Primer location based on human Norwalk M87661.

## Supplementary Materials

The following are available online at https://www.mdpi.com/1999-4915/12/5/490/s1, Figure S1: A phylogenetic tree of human sapovirus using 183 bp of partial RdRp region, Figure S2: Molecular Phylogenetic analysis of environmental sapovirus using 194 bp of the RdRp region, Figure S3: Phylogenetic tree of animal sapovirus based on partial RdRp (207 bp).
