# Supplementary material for: Distribution and Genetic Variability of Sapoviruses in Africa
Source: Viruses. 2020 Apr 27;12(5):490. doi: 10.3390/v12050490 (PMC7291139; doi:10.3390/v12050490)
Supplement: Supplementary file 1 [file viruses-12-00490-s001.pdf]

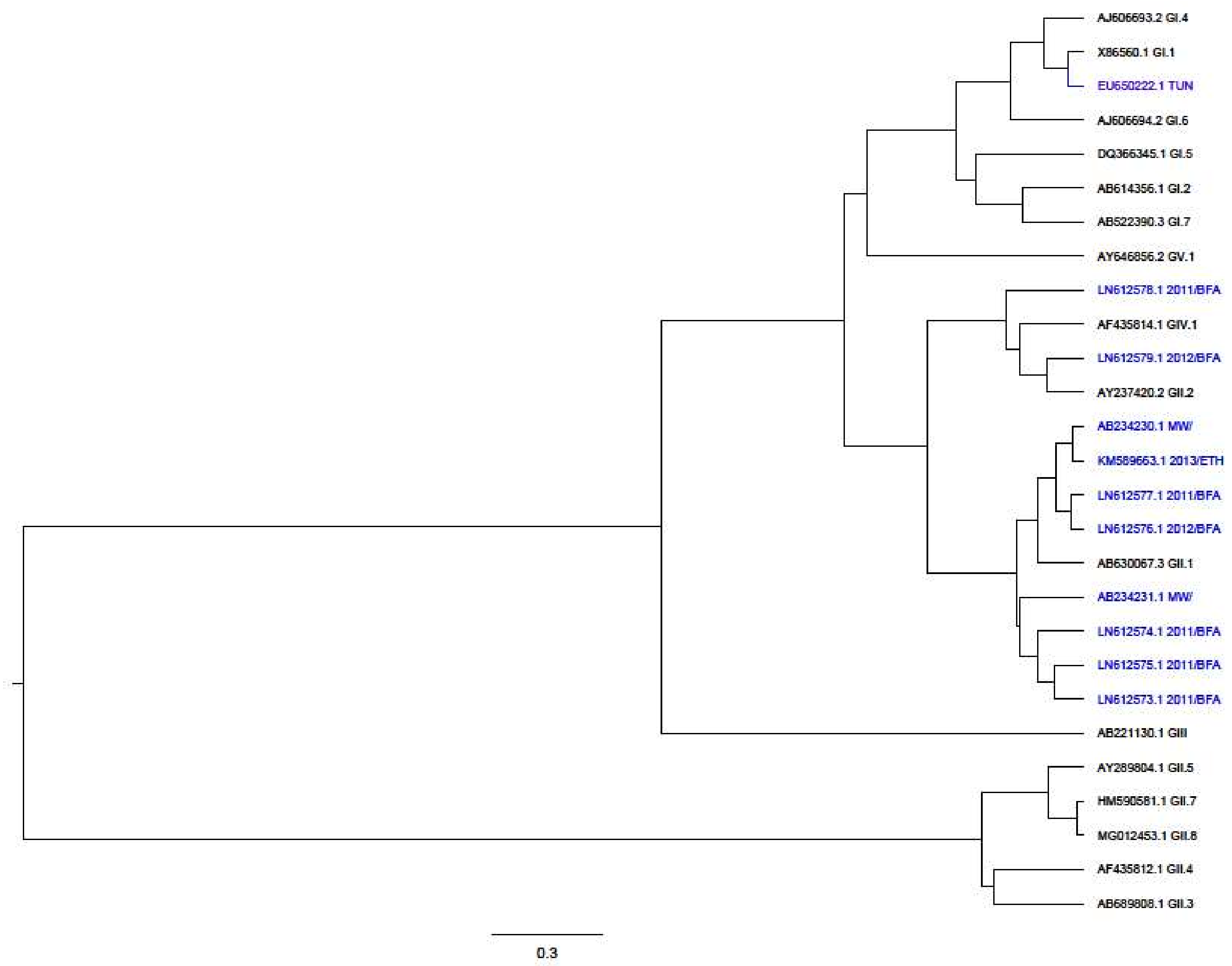

Figure S1. A phylogenetic tree of human sapovirus using 183bp of partial RdRp region. Reference sequences are in black font, sequences from reviewed papers are in blue. The evolutionary history was inferred by using the (BEAST v 1.10.4) Bayesian method, GTR model.

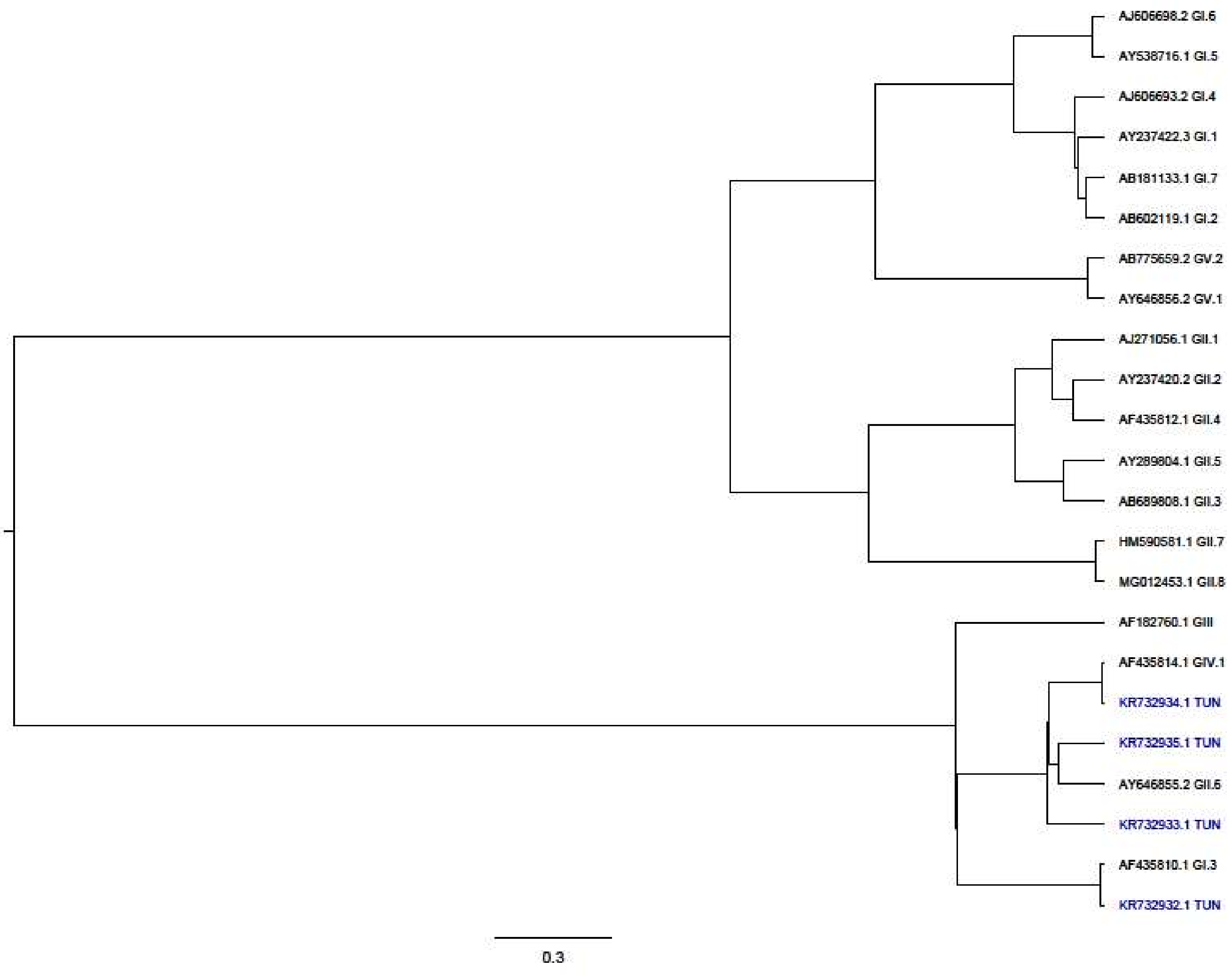

Figure S2. Molecular Phylogenetic analysis of environmental sapovirus using 194bp of the RdRp region .The evolutionary history was inferred by using the **(**BEAST v 1.10.4) Bayesian method, GTR model.

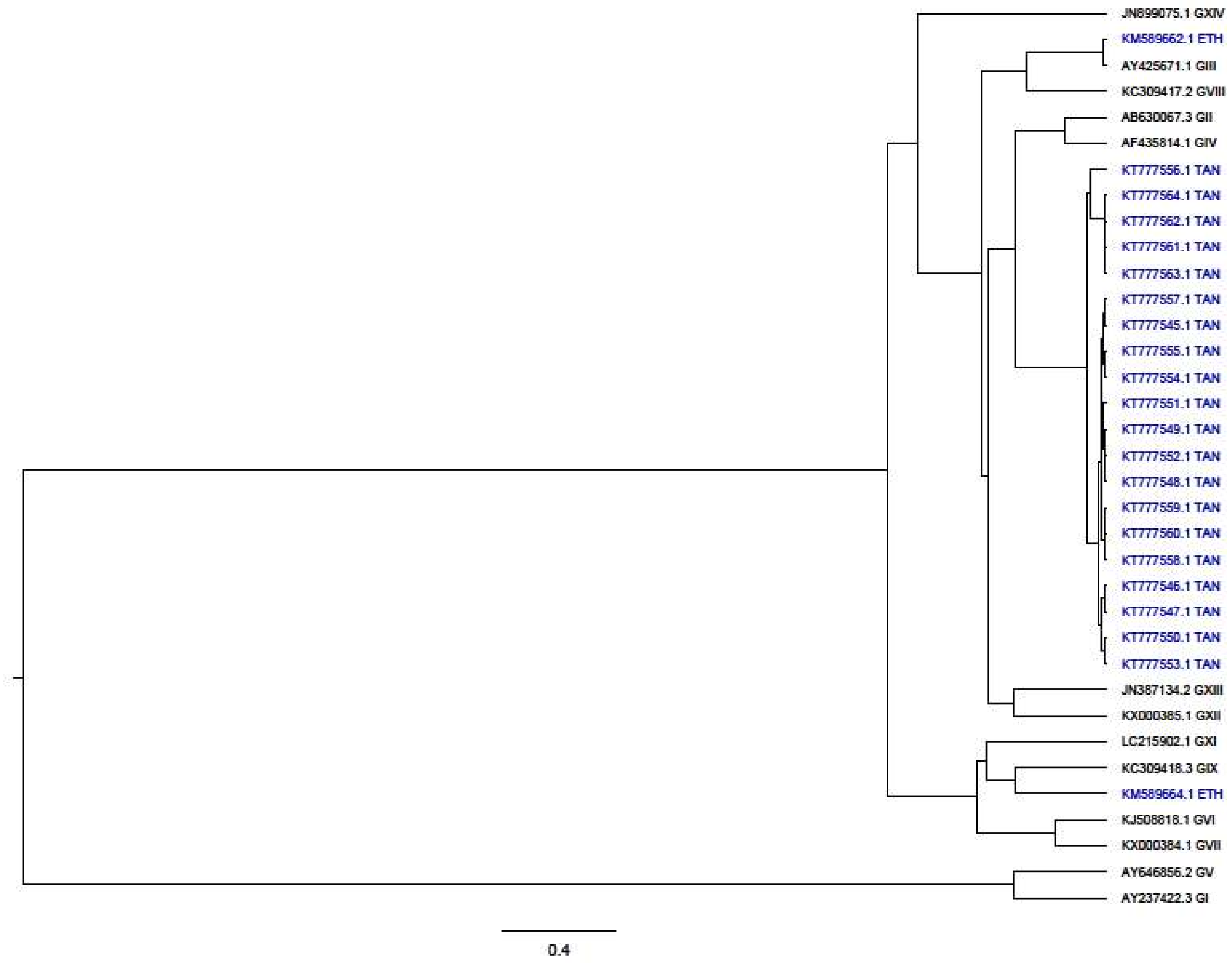

Figure S3. Phylogenetic tree of animal sapovirus based on partial RdRp (207 bp).The tree is constructed using (BEAST v 1.10.4) Bayesian inference method, GTR model.
